# Differences in Gait Characteristics of Patients with Lumbar Spinal Canal Stenosis (L4 Radiculopathy) and Those with Osteoarthritis of the Hip

**DOI:** 10.1371/journal.pone.0124745

**Published:** 2015-04-20

**Authors:** Noriaki Yokogawa, Yasumitsu Toribatake, Hideki Murakami, Hiroyuki Hayashi, Takeshi Yoneyama, Tetsuyou Watanabe, Hiroyuki Tsuchiya

**Affiliations:** 1 Department of Orthopedic Surgery, Graduate School of Medical Science, Kanazawa University, Kanazawa, Japan; 2 Department of Orthopedic Surgery, Kouseiren Takaoka Hospital, Takaoka, Japan; 3 School of Mechanical Engineering, College of Science and Engineering, Kanazawa University, Kanazawa, Japan; West Virginia University School of Medicine, UNITED STATES

## Abstract

It is important to differentially diagnose thigh pain from lumbar spinal stenosis (particularly lumbar fourth nerve root radiculopathy) and osteoarthritis of the hip. In this study, using a treadmill and a motion analysis method, gait characteristics were compared between these conditions. Patients with lumbar fourth nerve root radiculopathy had increased physiological knee flexion immediately after foot-ground contact, possibly owing to a slight decrease in the muscle strength of the quadriceps femoris muscle. Patients with osteoarthritis of the hip had decreased range of motion of the hip joint probably due to anatomically limited mobility as well as gait strategy to avoid pain resulting from increased internal pressure on the hip joint during its extension. Our facile and noninvasive method can be useful for the differential diagnosis of lumbar spinal canal stenosis from osteoarthritis of the hip.

## Introduction

Patients with pathogenic lesions of the hip joint frequently complain of pain at the anterior aspect of the thigh. However, fourth lumbar root (L4) entrapment may also manifest as radiating pain in the same region. Therefore, anterior thigh pain may be confused with pain originating in the hip. Offierski and MacNab caution experienced spine and hip surgeons that failure to recognize concurrent hip and spine disease, often called hip-spine syndrome, may lead to confusion, a mistaken diagnosis, or even erroneous treatment [1]. They also emphasize the necessity for ancillary investigation emphasizing the value of spinal nerve root infiltration or hip joint anesthetic injections in assessing the contribution of each area to the patient’s disability. Owing to the invasive nature of these methods, we have focused on walking motion analysis as a noninvasive alternative for differential diagnosis. The purpose of this study was to detect gait characteristics uniquely associated with L4 radiculopathy and with hip joint pain to help identify the main lesion in patients with hip-spine syndrome.

## Materials and Methods

### Ethics Statement

This study was approved by the ethics committee of Kouseiren Takaoka Hospital, and written informed consent for study participation was obtained from each patient.

### Subjects

Subjects included 29 individuals: 12 healthy volunteers (control group; four men, eight women; median age, 41.1 years; range, 25–55 years), 7 patients with lumbar spinal canal stenosis (LSS) and L4 radicular symptoms (L4 group; five men, two women; median age, 71.1 years; range, 56–80 years), and 10 patients with unilateral hip osteoarthritis (OA) (hip group; one man, nine women; median age, 65.7 years; range, 57–80 years). The healthy volunteers had no neurological or arthritic diseases causing gait disturbance. All subjects in the L4 and hip groups underwent surgery after gait analysis testing. Subjects were included in the L4 group if only the L4 nerve root was affected, as determined comprehensively using neurological testing, magnetic resonance imaging myelographic imaging, a nerve root block, and intraoperative findings. None of the subjects in the L4 group had decreased lower limb muscle strength on manual muscle testing (MMT). According to the Kellgren & Lawrence classification [2], the hip group consisted of two patients with grade 3 and eight patients with grade 4 hip OA. Cases with both L4 radiculopathy and hip OA and those with other gait abnormalities (e.g., knee OA) were excluded.

### Treadmill Protocol

TR20F II (SportsArt, Inc., Tainan, Taiwan, China) was used in this study. The experiment was performed on the treadmill at 0° of ramp incline. Free speed walking was used, i.e., walking speed in daily living. Measurements were discontinued if subjects were unable to walk owing to lower limb pain; those subjects without pain walked for 10 minutes. Handrail use while walking was limited to those cases at high risk of falls in order to avoid forward-bending position during gait. We prevented falls by standing behind the subject. MMT was performed before and after gait assessment.

### Measurement Methods

**Fig 1:**
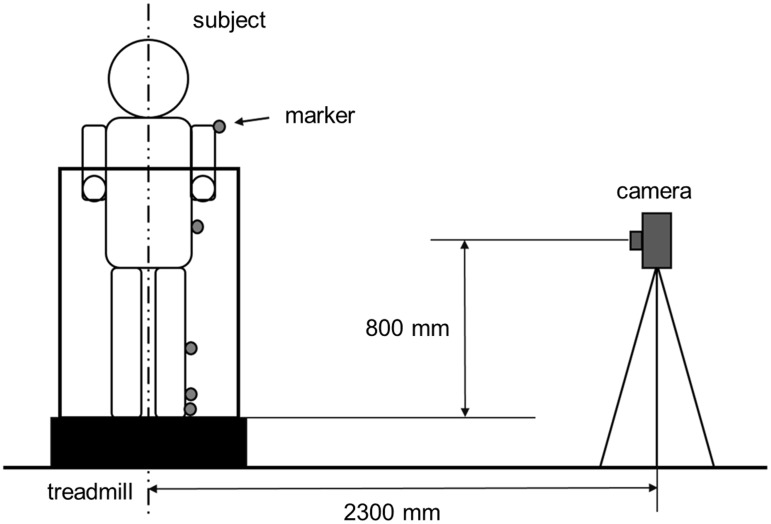
Walking measurement system. Light-emitting diode markers attached at the following sites: acromion, anterior superior iliac spine, fibular head, lateral malleolus of the ankle joint and the fifth metatarsal head. Walking assessed on a treadmill, and a digital camera recorded the walking motion.

We attached handmade, light-emitting diode markers on the affected side at 5 sites: acromion, anterior superior iliac spine, fibular head, lateral malleolus of the ankle joint, and the fifth metatarsal head. All subjects underwent examination of load walk on the treadmill using commercially available digital cameras to record walking motion in a dimly lit room (Fig 1). We performed the motion analysis for 10 seconds using our development program just prior to discontinuation of walking. The accuracy of this system depends on the resolution of the camera and the distance between the treadmill and camera, which was 0.007 ± 0.04 rad.

### Outcome Measure

**Fig 2:**
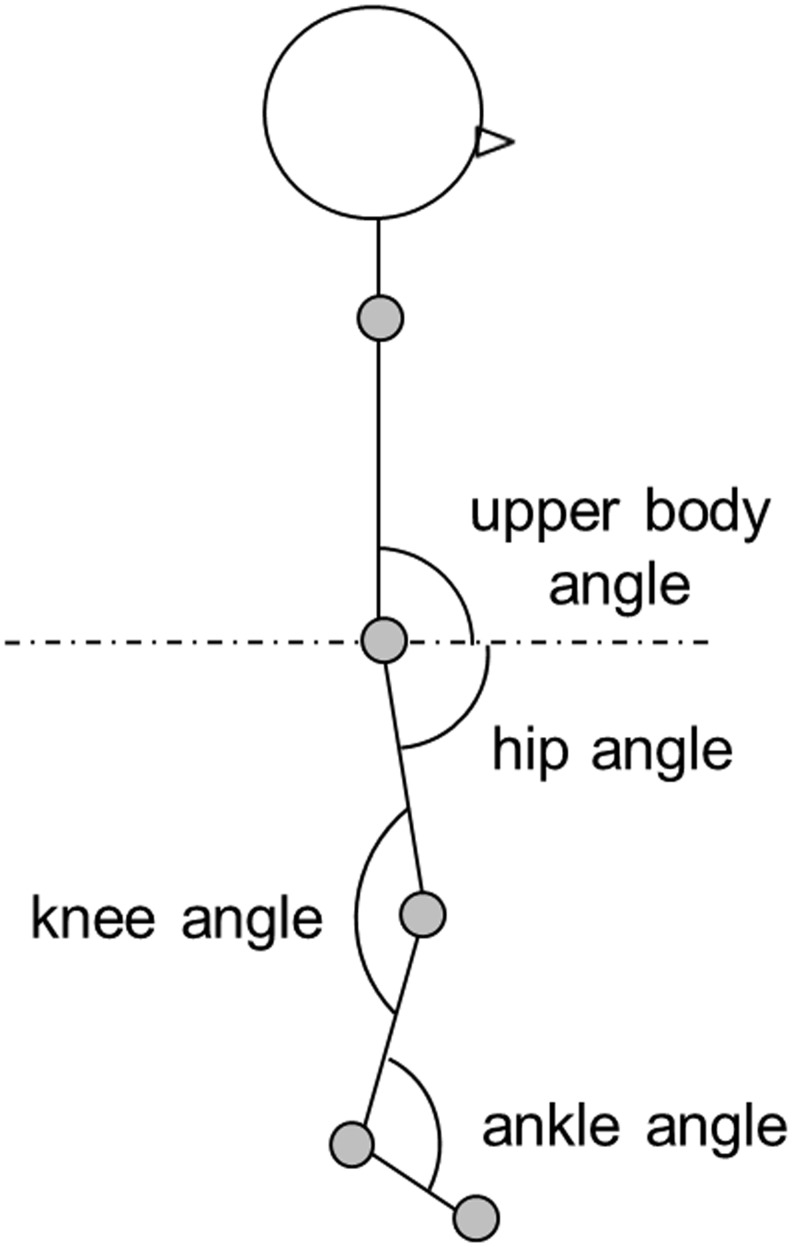
Sagittal plane view. The angle of each joint is defined in the schema.

Joint movement was visualized as a waveform, and the waveforms compared between the 3 groups. In this study, we focused on the motion of the lower limb and examined the hip and knee joint angles, defining each joint angle as shown in Fig 2.

### Statistical Analysis

Statistical evaluation was performed using Scheffe F-statistics for multiple comparisons by SPSS Statistical Software, version 19 (SPSS, Chicago, IL, USA). Statistical significance was set at a *P*-value less than 0.05.

## Results

Twenty-three subjects completed the 10-minute walking session on the treadmill: 12 subjects in the control group, 3 in the L4 group, and 8 in the hip group. None of the subjects in the control group, 2 subjects in the L4 group, and 5 subjects in the hip group used handrails while walking. None of the subjects had any changes in the MMT grades before and after walking.

### Waveform in Normal Gait

**Fig 3:**
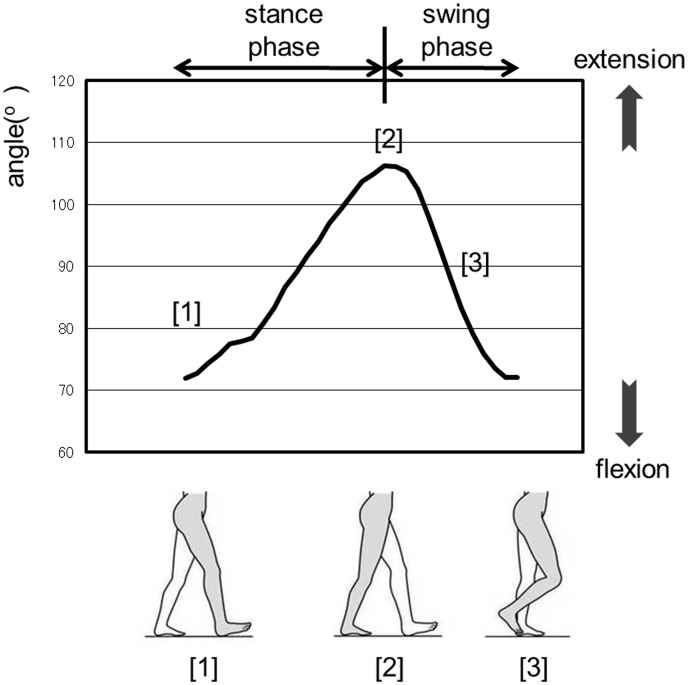
The waveform of the hip joint during normal gait.

**Fig 4:**
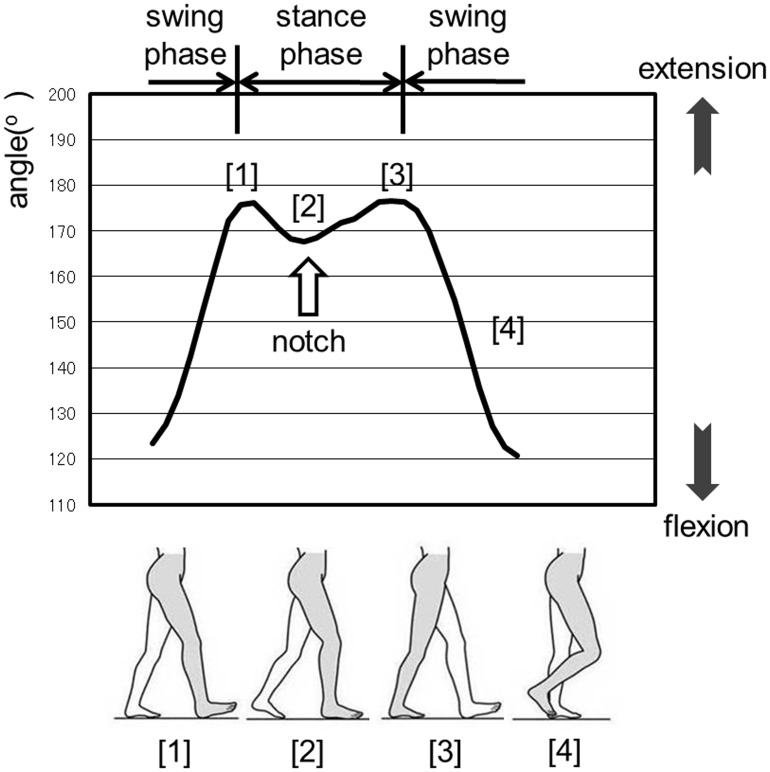
The waveform of the knee joint during normal gait.

The waveform in normal gait can be explained using the analysis results of the control group. As shown in Fig 3, the hip joint waveform shows a single-peak. The maximum flexion of the hip joint is established at the beginning of the stance phase, corresponding to the time of heel contact on the treadmill (Fig 3[1]). The hip joint gradually extends to reach maximum extension (Fig 3[2]) and subsequently, enters the swing phase (Fig 3[3]). As shown in Fig 4, the knee joint waveform traces out the letter “M”; in other words, the wave form contains 2 local maximum and one local minimum corresponding physiologically to 2 extension movements and a single flexion movement during the stance phase. The knee joint reaches its first local maximum with heel contact on the treadmill during extension; this movement corresponds to the beginning of the stance phase (Fig 4[1]). The knee joint then flexes for weight bearing, producing the local minimum; the local minimum knee flexion was recorded as a notch (Fig 4[2]). After that, the knee joint again extends to push the foot off the treadmill belt, yielding a second maximum (Fig 4[3]). Subsequently, the latter, large wave of flexion occurs during the swing phase (Fig 4[4]).

### Gait Characteristics

**Fig 5:**
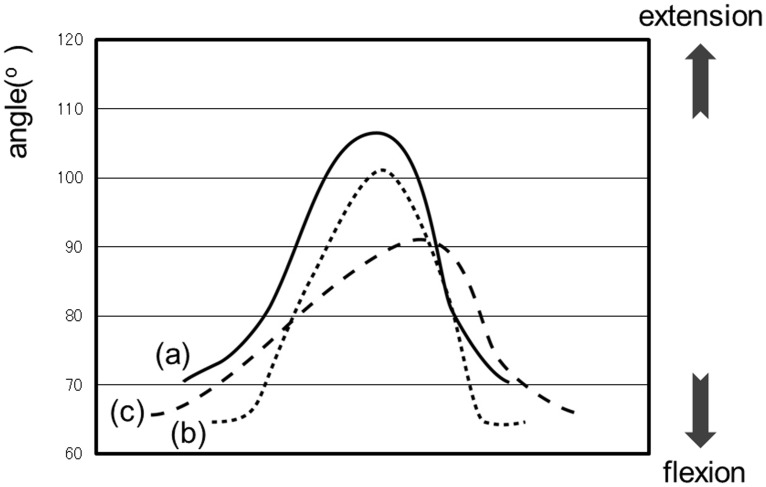
The hip joint waveform of a representative case for each group. a) control group: solid line, b) L4 group: dotted line, c) hip group: perforated line.

**Fig 6:**
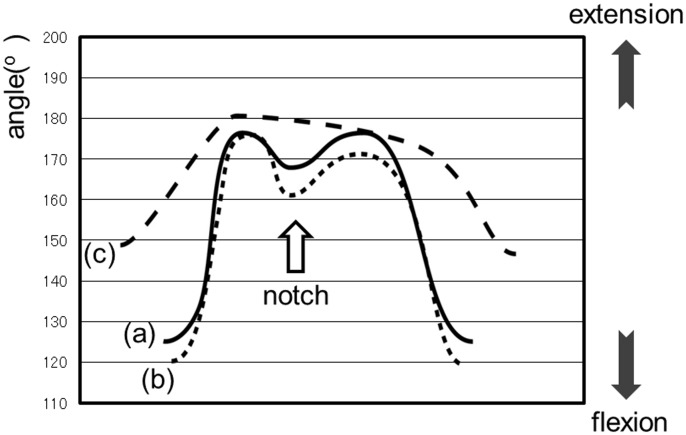
The knee joint waveform of a representative case for each group. a) control group: solid line, b) L4 group: dotted line, c) hip group: perforated line.

Data are presented as means ± standard deviations. Fig 5 shows the hip joint waveform of a representative case for each group. All three groups had similarly shaped waveforms. The amplitude of the waveform, which indicates the articular range of motion, was 35.7 ± 6.7° in the control, 26.1 ± 7.2° in the L4, and 18.3 ± 3.6° in the hip groups. The hip group had a significantly smaller mean amplitude than the other two groups. Fig 6 shows the knee joint waveform of a representative case in each group. In the knee joint of the L4 group, the waveform was similar to that of the control group; however, the knee extension at the initial foot contact was relatively greater than that at the second extension and the notch depth was larger than that of the other groups. In contrast, the notch depth was smaller in the waveform of the hip group than that of the control group. The notch disappeared in five of the 10 patients in the hip group, and their waveforms consisted of one peak with a curve sloping downward on the right side. The mean notch depth was 4.9 ± 3.8° in the control, 8.6 ± 6.7° in the L4, and 2.4 ± 3.2° in the hip groups. Thus, the L4 group had a significantly higher mean depth than the control group and the hip group had a significantly lower mean depth. The amplitude of the knee joint waveform (articular range of motion) was 61.6 ± 12.1° in the control, 49.1 ± 9.4° in the L4, and 37.0 ± 13.1° in the hip groups. The waveform of the hip group had significantly diminished amplitude compared to that of the control group. No significant differences in hip and knee joint movements were observed between the handrail user and non-handrail user groups.

## Discussion

Kinetic gait analysis was first used to analyze of normal gait by Murray et al. [3,4] and continues to be used in the evaluation of pathological gait with the aid of ever-advancing devices of measurement with improving precision. In recent years, gait analysis using a force plate has allowed multidimensional assessment using various temporal and spatial factors, as well as simultaneous electromyographic recording. On the other hand, more common use of the force plate is hindered because of the need for space, specialized equipment, high personnel and time costs, and complex data analysis. To increase the clinical use of the gait analysis method, we considered that ease of use, low cost, and simplified assessment items were necessary. Thus, this study used a simple motion analysis method and treadmill to quantitatively and visually examine gait characteristics by focusing on joint motions. Although the two-dimensional motion images produced by our method do not contain three-dimensional information, we found that our images provide enough data to adequately define the gait characteristics of diseases such as LSS and hip OA. One previous study reported that, although treadmill walking differs slightly from normal gait (including a slower comfortable walking speed on a treadmill), motion patterns in treadmill walking are almost equivalent to those in normal gait [5]. In this study, gait characteristics of the normal group were consistent with the results of studies that evaluated level walking [3,4,6]. The treadmill method has advantages, including the ability to analyze multiple continuous steps and increased safety using handrails for individuals with decreased waking ability. For these reasons, we considered it the most suitable method.

The characteristic finding of the L4 group was a “deeper notch” for the knee joint. When gait characteristics of neurogenic disease are evaluated from the kinematic and morphologic aspects, two mechanisms should be considered. The first mechanism is that spastic and flaccid paralysis affects the joint motion through muscles; this is an essential finding that strongly reflects the nature of the disease. Another mechanism is the secondary finding of unconsciously performed gait strategy such as an avoidance reaction for pain and a compensatory reaction for paralysis. The deeper notch was thought to be due to slightly decreased muscle strength of the quadriceps femoris innervated by the L4 nerve root, which has not been detected on MMT. In cases with quadriceps weakness, as gait strategy, knee hyperextension may occur to reduce the demand on a weak quadriceps at the foot—ground contact. That is, knee hyperextension at the foot—ground contact was observed as a deeper notch in this study. Suda et al. reported that abnormalities in gait style were noted in patients with neurogenic disease immediately after they began to walk [7]. In this study, no subject showed any difference in MMT grade before and after walking. This result suggested that patients have acquired a style of walking that precludes the appearance of symptoms, such as a compensatory reaction for paralysis. However, slight quadriceps muscle paralysis in the L4 group is merely a hypothesis. Verification of the muscle paralysis using a nerve conduction study, electromyography, and objective muscle strength testing (e.g., a tensiometer) is necessary to create a more validated study, which will be the objective of our future study.

In the hip group, the decrease in the hip range of motion was thought to involve not only anatomical factors (for example, osteophyte formation and thickening of the joint capsule), but also factors related to gait strategy to decrease pain. That is, patients with hip OA likely learned to limit the over-extension of the hip joint as a self-control reaction to avoid pain resulting from increased internal pressure of the hip joint. Murray et al. examined the decrease in the range of motion of the knee as a secondary finding to the decrease in the range of motion of the hip [8]. The hip joint begins full extension in the late stance phase, and the knee joint can be fully extended; however, if hip joint extension is limited, then the knee joint will remain flexed. Moreover, the decrease in knee joint extension in the late stance phase results in notch disappearance and a single-peak waveform with the curve sloping downward on the right side.

The present study has several limitations. First, the sample size was small. Our gait analysis had a simple design so that it could be easily conducted in daily practice. Therefore, compared to other precise and diversified gait analysis methods, it is difficult to determine detailed gait characteristics using our method. Thus, the reliability of each gait characteristic should be enhanced by the collection of more samples. Second, because the control group consisted of healthy volunteers recruited from our institution, they were younger than those in the disease groups. Although almost similar walking patterns between elderly and young people have been reported elsewhere [9], the analysis of healthy elderly subjects is necessary to increase the reliability of our research findings. In addition, the disease groups were biased because all patients were treated with surgery; thus, it is important to also consider patients with less severe disease who did not require surgery.

However, the present study is the first to demonstrate differences in gait characteristics of L4 radiculopathy and hip OA by using a very simple gait analysis method of treadmill and motion analysis. The findings obtained from this study can be useful for the differential diagnosis of and treatment decision making for hip—spine syndromes. With the aim of clinical application, we would like to similarly examine cases of both L4 radiculopathy and hip OA in the future to evaluate the effectiveness of this gait analysis method as a diagnostic tool.

## Conclusion

We developed a facile and noninvasive examination method for gait analysis and identified several useful factors for differentiating patients with L4 radiculopathy from those with hip OA.
